# Excitation and Inhibition Imbalance in Rett Syndrome

**DOI:** 10.3389/fnins.2022.825063

**Published:** 2022-02-18

**Authors:** Wei Li

**Affiliations:** Department of Neurobiology, University of Alabama at Birmingham, Birmingham, AL, United States

**Keywords:** excitation/inhibition balance, MeCP2, Rett syndrome, synaptic transmission, neurodevelopmental disorder

## Abstract

A loss of the excitation/inhibition (E/I) balance in the neural circuit has emerged as a common neuropathological feature in many neurodevelopmental disorders. Rett syndrome (RTT), a prevalent neurodevelopmental disorder that affects 1:10,000–15,000 women globally, is caused by loss-of-function mutations in the *Methyl-CpG-binding Protein-2* (*Mecp2*) gene. E/I imbalance is recognized as the leading cellular and synaptic hallmark that is fundamental to diverse RTT neurological symptoms, including stereotypic hand movements, impaired motor coordination, breathing irregularities, seizures, and learning/memory dysfunctions. E/I balance in RTT is not homogeneously altered but demonstrates brain region and cell type specificity instead. In this review, I elaborate on the current understanding of the loss of E/I balance in a range of brain areas at molecular and cellular levels. I further describe how the underlying cellular mechanisms contribute to the disturbance of the proper E/I ratio. Last, I discuss current pharmacologic innervations for RTT and their role in modifying the E/I balance.

## Introduction

Rett syndrome (RTT; OMIM identifier #312750), a neurodevelopmental disorder, is the leading cause of intellectual disabilities in girls in 1:10,000–15,000 births (Armstrong, 2005; Laurvick et al., 2006; Chahrour and Zoghbi, 2007). More than 95% of RTT cases are caused by loss-of-function mutations in the X-linked gene encoding a transcriptional regulator known as methyl-CpG-binding protein 2 (MeCP2). Individuals with RTT appear to develop typically until 6–18 months, when a constellation of neurological symptoms emerge, including autism-like behaviors, stereotypic hand movements, aberrant visual contacts, breathing irregularities, seizures, and loss of acquired speech (Hagberg et al., 1983; Neul et al., 2010).

More than two decades of intensive research in RTT has revolutionized our understanding of its pathogenesis since MeCP2 was discovered as a key culprit. It was first hypothesized that MeCP2 functions as a transcriptional repressor by targeting DNA at CpG inlands (Nan et al., 1997; Jones et al., 1998). Later findings suggest that MeCP2 also has a role in gene activation and exhibits widespread binding distribution across target genes (Chen et al., 2015; Horvath and Monteggia, 2018; Lee et al., 2020). MeCP2 is prevalently expressed in the brain and its deficiency in neurons was initially regarded as the exclusive cause of RTT (Chahrour and Zoghbi, 2007). However, later studies found that non-neuronal cells, including glia, have a pathological role in RTT etiology (Lioy et al., 2011). Furthermore, MeCP2 was thought to be only essential for brain development, but conditional mouse models of RTT show similar symptoms when MeCP2 is deleted during adulthood. These abnormalities can be reversed by re-expression of MeCP2 in adult mice (McGraw et al., 2011; Cheval et al., 2012; Robinson et al., 2012). These discoveries suggest that MeCP2 is not only required for brain development but also for maintaining intact neural function throughout the life span. In terms of the onset of symptoms, many subtle neurological aberrances in RTT patients occur earlier than neuroscientists initially thought, pointing out that the clinical manifestations seem to be a result of a gradual accumulation of brain pathology rather than a rapid deterioration (Einspieler et al., 2005; Marschik et al., 2013). In search of underlying cellular dysfunction, researchers started to acknowledge the loss of excitation/inhibition (E/I) balance as a fundamental mechanism underlying this disorder (Ip et al., 2018). Excitatory and inhibitory synapses undergo spatial and temporal modification during the postnatal development; E/I balance needs to be dynamically maintained for the neural circuit to function properly (Niciu et al., 2012; Oh and Smith, 2019). Understanding the cause and consequence of the unbalanced E/I in RTT is undoubtfully a crucial step for seeking or evaluating any therapeutic interventions.

In the first part of this review, I will elucidate the characteristics of two central E/I components in different brain areas: glutamatergic activity and GABAergic activity. I will also discuss several critical elements, which can pose a significant impact on E/I balance. The second part of this review will detail the consequence of E/I imbalance on brain function at the synaptic and network levels. Lastly, I will elaborate on several ongoing pharmacological treatments and their impact on E/I balance. Of note, although RTT mostly affects female individuals, the majority of animal studies use male *Mecp2* hemizygous knockout (KO) mice to investigate the consequence of *Mecp2* deletion on genetic, molecular, cellular, and circuit mechanisms. This choice takes into consideration the fact that the complete absence of MeCP2 in males will allow unambiguous interpretations of the observed results without the potential confounding contribution of wildtype (WT) cells in the mosaic brain of female *Mecp2* mice. However, more recent studies, especially those investigating therapeutic treatments, have adopted female heterozygous (HET) mice as they better recapitulate the pathogenesis of human RTT. Even though both genotypes share many similar pathophysiological manifestations, they do display some differences that are of significance for further basic and translational research. For detailed comparisons between two genotypes, I refer readers to a recent review (Ribeiro and MacDonald, 2020). In this discussion, to distinguish them wherever possible, I denote male *Mecp2*-deficient mice as *Mecp2* KO mice and female mice as *Mecp2* HET mice. Additionally, many mouse models have been utilized in RTT research. I will present abbreviated strain names if provided in the original articles; for the full strain names and their genetic background, see our previous review (Li and Pozzo-Miller, 2012).

## Brain Region-Specific Loss of Excitation/Inhibition Balance

Studies in the entire brain have been used to determine E/I imbalance in RTT. For example, immunoblotting assay shows that expressions of gamma aminobutyric acid type A receptor (GABA_*A*_R) subunit β3 and GABA_*B*_R2 are reduced in brain tissues of RTT patients and many MeCP2-deficient mouse models (Samaco et al., 2004; Nguyen et al., 2012). As measured by magnetic resonance spectroscopy (MRS), the mean glutamate is larger in RTT patients than controls (Horská et al., 2009). By contrast with the higher glutamate concentration, whole-brain homogenates prepared from *Mecp2* KO mice (Bird line) show reductions in two glutamate receptors (GluRs) aminomethylphosphonic acid receptor (AMPAR) and *N*-methyl-D-aspartate receptor (NMDAR), and in two synaptic vesicle proteins glutamate transporter (vGlut) and synapsin (Nguyen et al., 2012). Furthermore, the expression of NMDAR subunits GluN1 and GluN2A is increased, but that of GluN2B is unchanged in *Mecp2* KO mice (Bird line) (Maliszewska-Cyna et al., 2010). These incongruities are likely as a study using c-fos expression as a surrogate of neuronal activity shows that the whole-brain activity is not homogenous, with the forebrain tending to be hypoactive and the hindbrain being hyperactive (Kron et al., 2012). Considering this concept, I will discuss the E/I imbalance below in a brain region-specific fashion (Table 1). Even in the same brain region, different types of cells may have a drastically distinct role in E/I balance. For instance, mice lacking MeCP2 in somatostatin-positive (SOM+) interneurons develop seizures but display normal memory and social interaction; in contrast, mice lacking MeCP2 in parvalbumin-positive (PV+) interneurons do not exhibit epileptic activity but rather show cognitive deficits (Ito-Ishida et al., 2015). Additionally, various mutations in RTT patients and many different RTT animal models are analyzed, which could account for some controversies. If needed, these aspects will be mentioned in the following discussions.

**TABLE 1:** Excitation/inhibition (E/I) imbalance in the different brain regions of RTT patients or mice.

Brain Region		Species	E/I feature	References
Cerebral cortex	Motor	RTT patients	↓ muscle response, ↓ GABAergic inhibition	Eyre et al., 1990; Bernardo et al., 2020
		*Mecp2* KO^Jae^	↓ eEPSC (PCs, L5), ↑ PV+ INs	Tropea et al., 2009; Morello et al., 2018
		*Mecp2* KD	↓ Glu uncaging eEPSCs, → intrinsic, → eIPSCs (PCs, L2/3)	Wood et al., 2009
		*SD-Mecp2* KO	↓ Muscimol-induced spike rate	Li et al., 2021
	Somatosensory	*Mecp2* KO^Jae^	↑ PV+ INs; ↑ sIPSC charge, ↓ eEPSC charge, ↓ synapses (PCs, L5)	Dani et al., 2005; Dani and Nelson, 2009; Morello et al., 2018
		*Mecp2* cKO (INs)	↓ GABA, ↓mIPSC amplitude (PCs, L2/3)	Chao et al., 2010
		*Mecp2* cKO (f-INs)	→ sIPSCs (PCs, L5)	Zhang et al., 2014
		*Mecp2* cKO (f-PCs)	→ eIPSCs, ↓ synapses, ↓ sIPSCs (PCs, L5)	Zhang et al., 2014
	Prefrontal	*Mecp2* KO^Jae^	↓ eEPSC, → eIPSPs, ↓ synapse density, → PV, ↓ NMDAR but ↑ GluN2B, ↑ hippocampal input to PV+ INs	Sceniak et al., 2016; Howell et al., 2017; Phillips et al., 2019
		RTT patients/*Mecp2* KO*^Bird^*	↑ (early), ↓ (later) NMDAR	Blue et al., 1999; Blue et al., 2011
	Visual	*Mecp2* KO*^Bird^*	↑ GluN2A/2B (early in PV+ INs, but later in PCs), → AMPAR, ↑ PV, ↑ GAD67 and 65, ↑ sIPSC (INs)	Lee et al., 2008; Durand et al., 2012; Krishnan et al., 2015; Mierau et al., 2016
			↓ visual EPSCs and IPSCs, but ↑ ratio	Banerjee et al., 2016
		*Mecp2* KD	↓ AMPAR	Blackman et al., 2012
		*Mecp2* cKO (INs)	↑ intrinsic, ↓ sEPSCs, → spikes (PV+ INs)	He et al., 2014
	Auditory	*Mecp2* HET*^Bird^*	↑ PV, ↓ disinhibition (PCs)	Krishnan et al., 2017; Lau et al., 2020a
Hippocampus	All	Cultured neurons	↓ mEPSC frequency, → mEPSC amplitude, ↑ eEPSCs	Nelson et al., 2006; Nelson et al., 2011
		Autaptic neurons	↓ synapses, ↓ mEPSC frequency, ↓ eEPSCs	Chao et al., 2007
		*Mecp2* KD neurons	↓ synapses, ↓ mEPSC frequency	Ma et al., 2015
	CA1	*Mecp2* KO^Jae^	↑ field EPSPs, ↑ eEPSCs	Calfa et al., 2011; Li et al., 2016
			→ field EPSPs, ↓ GluN2A/2B	Asaka et al., 2006
		*Mecp2* KO^Bird^	↑ extracellular Glu	Balakrishnan and Mironov, 2018a,b
		*Mecp2* ^308^	↑ field EPSPs	Moretti et al., 2006
		*Mecp2* *^stop^*	→ intrinsic, → PPR	McLeod et al., 2013
		*Mecp2* cKO (astrocytes)	↓ tonic GABA currents	Dong et al., 2020
	CA3	*Mecp2* KO^Jae^	↑ mEPSCs amplitude (PCs), ↓ mIPSC amplitude (PCs), → IN density and intrinsic, ↓ mIPSC (INs)	Calfa et al., 2015
		*Mecp2* KO^Bird^	↓ sEPSC frequency, ↑ sIPSC frequency	Zhang et al., 2008
			↓ (later) sIPSC amplitude and frequency	Lozovaya et al., 2019
			→ GABAergic activity	Belaïdouni et al., 2021
Brainstem	Ventrolateral medullar	*Mecp2* KO^Bird^	↓ sIPSC amplitude and frequency, ↑ sEPSC amplitude and frequency, ↓ GABA, ↓ GABA transporter, ↓ GABA_*A*_R, → glycinergic	Medrihan et al., 2008; Abdala et al., 2010
	nTS	*Mecp2* KO^Jae^	↑ mEPSC and sEPSC amplitude	Kline et al., 2010
		*Mecp2* KO^Bird^	↓ sIPSC amplitude and frequency, ↓ mIPSC amplitude, ↓ eIPSCs	Chen et al., 2018
	XII and DMNV nuclei	*Mecp2* *^R168X^*	↓ GABAergic and glycinergic	Xing et al., 2021
	LC	*Mecp2* KO/HET^Jae^	↑ intrinsic	Taneja et al., 2009
		*Mecp2* KO^Bird^	↓ GABA releases, ↑ extrasynaptic GABA_*A*_R subunits δ and α6	Jin et al., 2013; Zhong et al., 2015
Others	Cerebrospinal fluid	RTT patients	↑ Glu	Hamberger et al., 1992
	Amygdala	*Mecp2* ^308^	↓ eEPSCs and PPR reduction	Gambino et al., 2010
	dLGN	*Mecp2* KO^Bird^	↓ eEPSCs	Noutel et al., 2011

*For detailed description, see the text. nTS, nucleus tractus solitarius; LC, locus coeruleus; dLGN, dorsal lateral geniculate nucleus; Jae, Jaenisch line; IN, interneuron; f-INs, forebrain interneurons; f-PCs, forebrain pyramidal cells; PV, parvalbumin; PC, pyramidal cell; Glu, glutamate. SD-Mecp2 KO, Sprague Dawley rat model of Mecp2 knockout.*

### Excitation/Inhibition Imbalance in the Cerebral Cortex

One of the first studies was to test neural activity in the motor cortex of human RTT subjects (Eyre et al., 1990). This study demonstrates that muscle responses are normal with electromagnetic stimulation in the cervical spinal cord, whereas they have a low threshold, a short latency, and a protracted duration, suggesting hyperactivity in the motor cortex. The propensity for neural excitation in this brain region was recently confirmed in a larger sample of RTT patients. This study postulates that the increased excitability is caused by dysfunction of GABAergic activity (Bernardo et al., 2020). On the contrary, animal studies in the sensorimotor cortex of *Mecp2* KO mice (Jaenisch line) show that evoked excitatory postsynaptic currents (eEPSCs) are reduced in pyramidal neurons of layer 5 (Tropea et al., 2009). This reduction is associated with the postsynaptic deficit, as the amplitude of spontaneous EPSCs (sEPSCs) and immunoreactivity for postsynaptic density protein 95 (PSD95) are both decreased. However, it does not exclude the possibility of presynaptic impairment, because action potential-dependent sEPSCs also rely on presynaptic activity. Immunohistochemical assay for a presynaptic marker could lead to a decisive conclusion. In a different study, examination of Glu uncaging-evoked EPSCs in layers 2/3 of the motor cortex demonstrates that synaptic input from layers 3/5A is reduced following RNA interference-mediated *Mecp2* knockdown (KD) in pyramidal neurons (Wood et al., 2009). The topographic distribution of synapses in these layers remains intact; intrinsic properties and inhibitory inputs are also normal in these MeCP2-deficient neurons. Nevertheless, PV+ interneurons that provide the GABAergic inhibition in the motor and somatosensory cortices are increased in density, resulting in a reduced network activity as measured by voltage-sensitive dye imaging (Morello et al., 2018). Consistent with this result, multi-unit recordings in the motor cortex reveal an increased synaptic inhibition in a rat model of RTT (Li et al., 2021). Collectively, more studies in the motor cortex are needed to resolve the discrepancy of findings in human RTT and animal models.

In the somatosensory cortex, the number of PV+ interneurons is increased (Morello et al., 2018). Congruently, spontaneous inhibitory postsynaptic currents (sIPSCs) in pyramidal neurons are augmented in charge transfer (Dani et al., 2005). Together with the increased inhibitory activity, the decreased charge of eEPSCs contributes to a reduction of neuronal discharge in layer 5 pyramidal neurons. The same group further demonstrates that the decreased excitatory neurotransmission is caused by the low number of synaptic contacts (Dani and Nelson, 2009). Furthermore, the amplitude and frequency of action potential-independent miniature (m)EPSCs are decreased but those of mIPSCs are normal, indicating that pre- and postsynaptic machinery is affected in the excitatory but not the inhibitory synapses (Dani et al., 2005). Compared to these results obtained from constitutive *Mecp2* KO mice (Jaenisch line), *Mecp2* deletion solely in GABAergic interneurons results in lower GABA content and smaller mIPSCs in layers 2/3 pyramidal neurons (Chao et al., 2010). Restoring MeCP2 expression in GABAergic interneurons alone is sufficient to alleviate these molecular and cellular deficits found in constitutive *Mecp2* KO mice (Ure et al., 2016). However, forebrain-specific *Mecp2* deletion from interneurons does not affect sIPSPs in layer 5 (Zhang et al., 2014); it may affect other aspects of synaptic transmission because these animals display some RTT-like features (Chao et al., 2010). Interestingly, selective deletion of *Mecp2* from excitatory neurons has no effect on excitatory transmission but instead reduces inhibitory synapse numbers and neurotransmission in the somatosensory cortex and the prefrontal cortex (Zhang et al., 2014). Altogether, the different results obtained from constitutive and conditional KO mice suggest the possible compensation during the development.

In *Mecp*2 KO mice, the prefrontal cortex shows an amplitude reduction in eEPSCs but normal eIPSCs, which is consistent with the reduced density of excitatory synapses and normal PV expression (Sceniak et al., 2016; Howell et al., 2017). The total levels of NMDARs are decreased, but the relative fraction of one subunit, GluN2B, is conversely increased. In RTT patients and *Mecp2* KO mice (Bird line), NMDAR expression in the prefrontal cortex also declines at a later age following an initial increase (Blue et al., 1999; Blue et al., 2011). In addition to the cortical origin, hypoactivity in the prefrontal cortex may be partially caused by hyperactive hippocampal input that preferentially targets PV+ interneurons in *Mecp2* KO mice (Jaenisch line) (Phillips et al., 2019).

In the visual cortex, the E/I balance has been extensively investigated in MeCP2-relevant research. An early study shows that pharmacological blockade of MeCP2-mediated inhibition of GluN2B transcription enhances its expression (Lee et al., 2008), implying that GluN2B expression is likely to be increased in *Mecp2* KO mice. Indeed, it is true in excitatory neurons of the visual cortex. The continuous GluN2B expression in pyramidal neurons disrupts normal developmental replacement of GluN2B by GluN2A (Durand et al., 2012; Mierau et al., 2016). However, in contrast to the delayed increase in the GluN2A/GluN2B ratio in excitatory neurons, the increasing ratio is instead accelerated in PV+ interneurons in the MeCP2-deficient visual cortex. This impairment is associated with vision regression, which can be reserved by early visual deprivation or genetic reduction of GluN2A in PV+ interneurons (Durand et al., 2012; Mierau et al., 2016). As opposed to NMDAR subunits, developmental modification of AMPAR composition remains unaffected in *Mecp2* KO mice (Bird line). The normal expression of AMPARs may be due to developmental compensation because *Mecp2* KD can reduce the number of AMPAR subunits GluA1 and GluA2 without affecting their accumulation in spared synapses (Blackman et al., 2012). The early increase in the GluN2A/GluN2B ratio results in an excessive innervation of PV+ interneurons on pyramidal neurons (Durand et al., 2012). Such hyperconnectivity seems to be unique to PV+ interneurons because transcription of the markers of other interneurons, such as calretinin and calbindin, are somewhat decreased. Furthermore, expression levels of PV and GABA synthetic enzymes glutamic acid decarboxylase 67 (GAD67) and GAD65 are increased, but the interneuron density remains unaltered (Krishnan et al., 2015). The precociousness of PV+ interneurons is consistent with the increased sIPSC amplitude observed in adjacent interneurons. Network activity was also found to be decreased. However, whether the increased inhibitory innervation among interneurons contributes to the reduced network activity remains to be determined; whether the electric coupling of interneurons is stronger is also an open question. These alterations result in an early onset of the experience-dependent critical period in the visual cortex, which can be rescued by genetically decreasing GAD67 levels (Krishnan et al., 2015). On the other hand, reduced PV expression was observed in mice with *Mecp2* deletion specifically in PV+ interneurons. This downregulation was also suggested to be responsible for the alteration of an experience-dependent critical period in the visual cortex. This deficit can be restored by the pharmacological enhancement of GABAergic activity (He et al., 2014). MeCP2-deficient PV+ interneurons exhibit increased membrane excitability but receive less excitatory input from pyramidal neurons, which results in an overall unaltered number of spontaneous spikes in PV+ interneurons. However, a different study shows that visually-driven excitatory and inhibitory potentials in pyramidal neurons are both decreased, but to a different degree, and the overall E/I ratio is increased in *Mecp2* KO mice (Bird line) (Banerjee et al., 2016). Again, these cellular alterations appear to be specific to PV+ interneurons, as no or fewer effects are observed in *Mecp2* deletion in SOM+ interneurons (He et al., 2014; Banerjee et al., 2016). However, it may not always be the case across the life span. A study in the visual cortex at different ages shows that although the intensity of PV immunostaining increases with age, but that of SOM and calbindin gradually decreases in *Mecp2* KO mice (Bird line) (Patrizi et al., 2020).

Few studies have been focused on the auditory cortex. In female *Mecp2* HET mice (Bird line), an altered inhibitory network, accompanied by increased expression of PV, can be restored by genetical reduction of GAD67 (Krishnan et al., 2017). Behaviorally, the disinhibition of deep-layer pyramidal neurons in response to maternal experience is impaired in *Mecp2* HET mice (Bird line) (Lau et al., 2020a).

### Dysfunctional Excitation/Inhibition Balance in the Hippocampus

An early study using cultured hippocampal neurons finds that the frequency of mEPSCs is decreased in *Mecp2* KO neurons (Bird line), whereas the amplitude of mEPSCs remains unchanged (Nelson et al., 2006). However, eEPSCs are larger in amplitude in the same condition (Nelson et al., 2011). These results suggest that spontaneous and evoked neurotransmission are distinctly impacted by MeCP2 deficiency. In contrast, in autaptic hippocampal neurons of *Mecp2* KO mice, the number of synapses is decreased, resulting in lower frequency of mEPSCs and smaller eEPSCs (Chao et al., 2007). Using shRNA to silence *Mecp2* in hippocampal neuron cultures, a study shows fewer synaptic contacts, associated with decreased mEPSC frequency and synaptic network activity (Ma et al., 2015). It is worth noting from this study that reduced spontaneous spikes are more synchronous and MeCP2-deficient neurons intrinsically fire more action potentials.

In addition to neuronal cultures, acute hippocampal slices are more often utilized to test excitatory and inhibitory activity; many different results have been reported by using different mouse models. In slices prepared from symptomatic *Mecp2* mice (308/Y line), which express a truncated form of MeCP2, increased excitatory synaptic transmission was observed in CA1 (Moretti et al., 2006). Voltage sensitive dye imaging in hippocampal CA1 of *Mecp2* KO mice (Jaenisch line) shows enhanced peak amplitude and more spread of voltage signals, consistent with the increased slope of the input-output relationship of field EPSPs or eEPSCs (Calfa et al., 2011; Li et al., 2016). In these *Mecp2* KO mice, the increase in amplitude and frequency of mEPSCs is shown in CA1, suggesting that both pre and postsynaptic sites are dysfunctional (Li et al., 2016). The increased postsynaptic response can be reflected by higher levels of GluA1. Consistent with the higher excitatory neuronal activity, a measurement of ambient glutamate with a fluorescence sensor expressed in pyramidal neurons discloses repetitive glutamate transients in CA1 of *Mecp2* KO mice (Bird line) (Balakrishnan and Mironov, 2018a,b). In contrast to these findings, a different group did not detect altered basal neurotransmission, nor changes in levels of GluA1, GluR2, and PSD95 in *Mecp2* KO mice (Jaenisch line) (Asaka et al., 2006). In hippocampal CA1 of *Mecp2* (stop/y) mice, where a stop cassette is inserted to abolish MeCP2 function, recordings show normal basal synaptic transmission but are more susceptible to higher gamma frequency oscillations and epileptiform bursts in response to excitatory challenging (McLeod et al., 2013). Impairment of NMDAR-mediated activity is also likely to have a significant role in altering excitatory synaptic transmission in CA1. A report shows decreased GluN2A expression and enhanced GluN2B expression in *Mecp2* KO mice (Jaenisch line) (Asaka et al., 2006). Excessive activation of extrasynaptic NMDARs is induced by abnormal Ca^2+^ rises in *Mecp2* KO astrocytes (Dong et al., 2018). Regarding the inhibitory component in the E/I relationship, there seems to be no changes in the density of GABAergic interneurons, the quantity of synaptically released GABA, and the level of GABA_*A*_R expression in CA1 of *Mecp2* KO mice (Jaenisch line) (Dong et al., 2020). However, CA1 shows low levels of tonic GABA resulting from enhanced activity of astrocytic GABA transporters. The reduced tonic GABA currents contribute to network hyperactivity. Besides alterations of excitation and inhibition alone, evidence also shows that reduced recurrent inhibition contributes to synaptic impairment. The decreased inhibition is due to a reduced excitatory synaptic drive onto interneurons (Lu et al., 2016). Consequently, hypersynchrony is generated, interfering with ripple oscillation-dependent memory consolidation in *Mecp2* HET mice (Bird line) (Kee et al., 2018).

In hippocampal CA3 of *Mecp2* KO mice (Bird line), the frequency of sEPSCs is decreased but that of sIPSPs is increased, causing a reduction in the frequency of spontaneous rhythmic field potentials (Zhang et al., 2008). However, in another study in hippocampal CA3 of *Mecp2* KO mice (Jaenisch line), it was found that the amplitude of mEPSCs is enhanced while that of mIPSCs is decreased (Calfa et al., 2015). This E/I imbalance is congruent with the biochemical evidence showing higher GluA1 puncta intensity and lower intensity of GABA_*A*_R α-1 subunits on pyramidal neurons. The density and intrinsic property of several types of interneurons are normal, whereas the excitatory drive onto these interneurons is impaired, as reflected by smaller and less frequent mEPSCs in interneurons. In an independent study using presymptomatic *Mecp2* KO mice (Bird line), the amplitude and frequency of sEPSC are increased, while those of sIPSCs are normal at birth (Lozovaya et al., 2019). However, at P15 the amplitude of sEPSCs is still high, but the frequency and amplitude of sIPSCs are reduced. Such developmental alteration of the E/I ratio accounts for synchronized population bursts in CA3. In contrast to these findings of reduced inhibitory activity in *Mecp2* KO mice, a recent study in CA3 of presymptomatic (P30–35) and symptomatic (P50–60) mice (Bird line) did not find evident changes in GABA_*A*_R-mediated activity, which suggests profound developmental compensation may occur (Belaïdouni et al., 2021).

It is certain that the E/I balance is lost in hippocampal CA1 and CA3 in *Mecp2* KO mice. The dentate gyrus (DG), one of the important subregions in the hippocampus that serves as a gate for information flow, has not been studied in the context of RTT (Jedlicka et al., 2018). How the integrity of excitatory and inhibitory transmission is sabotaged in DG is an important subject for future investigation.

### Excitation/Inhibition Perturbations in the Brainstem

Many investigations have been focused on the role of E/I imbalance in the brainstem of MeCP2-deficient mice. An early study using *in vitro* heart-brainstem preparations to study breathing inspiration and expiration finds that *Mecp2* KO mice (Bird line) exhibit irregular nerve activities, breathing arrhythmias, and apnea (Stettner et al., 2007). Injecting glutamate into the Kölliker-Fuse nucleus of the brainstem evokes a longer apnea in *Mecp2* KO mice, suggesting a critical role of excitatory neurotransmission in regulating respiratory activity. A recent report further shows that in a rat *Mecp2* KO model, excitatory activity in both nuclei responsible for inspiration and expiration is increased, which can be corrected by boosting GABAergic inhibition (Wu et al., 2021). However, the exact role of this cellular impairment in breathing abnormalities is unknown.

In the ventrolateral medullar brainstem, the amplitude and frequency of sIPSCs are decreased but those of sEPSCs are increased in *Mecp2* KO mice (Bird line), resulting in an overturn of E/I balance (Medrihan et al., 2008). The biochemical analysis of GABAergic synapses shows that GABA content, vesicular inhibitory transmitter transporter, and postsynaptic GABA_*A*_R subunits are downregulated. Thus, treatments that increase GABA activity and decrease glutamatergic activity have been shown to ameliorate irregular breathing patterns in *Mecp2* HET mice (Bird line) (Abdala et al., 2010).

The nucleus tractus solitarius (nTS) is situated in the brainstem, which conveys visceral afferent inputs to the autonomic pathway and thereby is important for the regulation of breathing. Neurons in the nTS of *Mecp2* KO mice (Jaenisch line) show increased amplitudes of mEPSCs and eEPSCs and are prone to spike discharge following afferent stimuli (Kline et al., 2010). Measuring GABA_*A*_R-mediated inhibitory activity in *Mecp2* KO mice (Bird line) shows that the amplitude and frequency of sIPSCs are reduced in *Mecp2* KO neurons (Chen et al., 2018). The amplitudes of mIPSCs, eIPSCs, and GABA_*A*_R agonist-induced currents are similarly decreased. Interestingly, extrasynaptic GABA_*A*_R subunits are conversely increased; however, the role of shifting inhibitory activity from synapses to extrasynapses is unknown in regulating E/I balance.

In the hypoglossal nucleus (XII) and dorsal motor nucleus of the vagus (DMNV), inhibitory action on neurons is mediated by both GABAergic and glycinergic inputs. These inhibitory functions are both decreased in *Mecp2* point mutation mice (R168X) (Xing et al., 2021), which is different from normal glycinergic activity in the ventrolateral medullar brainstem of *Mecp2* KO mice (Bird line) (Medrihan et al., 2008).

In addition to those brainstem nuclei required for controlling breathing, another brainstem region, the locus coeruleus (LC) has also been investigated in MeCP2-related research. LC is the primary site for norepinephrine (NE) synthesis, involved in stress responses. In *Mecp2* KO or HET mice (Jaenisch line), LC neurons are deficient in the NE-synthesizing rate-liming enzyme tyrosine hydroxylase (TH) and show hyperexcitable intrinsic properties (Taneja et al., 2009). Neuronal firing in the LC can be inhibited by GABA_B_R- and GABA_A_R-mediated actions on pre-and postsynaptic sites, respectively. In *Mecp2* KO mice (Bird line), both inhibitory mechanisms are impaired, resulting in decreased GABA release and lower activation to postsynaptic receptors (Jin et al., 2013). As in the nTS (Chen et al., 2018), extrasynaptic GABA_*A*_R subunits, including δ and α6, are increased in the LC (Zhong et al., 2015). The enhancement was suggested to be a compensatory reaction to the decreased GABAergic action in synapses. Pharmacological activation of these subunits can tone down the activity to a level similar to controls. In contrast to the other brain regions, it appears that the inhibitory activity was unanimously found to be decreased in the brainstem.

### Alterations of Excitation/Inhibition Balance in Other Brain Regions of Rett Syndrome

Excitation/inhibition imbalance is also manifested in many other brain regions. High levels of glutamate are found in the cerebrospinal fluid of RTT patients (Hamberger et al., 1992). The amygdala receives inputs from many brain regions including the cerebral cortex (Berretta, 2005). During synaptic elimination and maturation of the early development, the amplitude of EPSCs and paired pulse ratio (PPR) decline in cortico-amygdala synapses (Gambino et al., 2010). In *Mecp2* KO mice (308/Y line), the decrease ratio becomes more considerable. It remains to be addressed regarding the role of these alterations in contributing to E/I imbalance for this particular and other inputs to the amygdala. In the visual pathway, retinal ganglion cells (RGCs) send excitatory output to the dorsal lateral geniculate nucleus (dLGN) in the thalamus. During the visual critical period, levels of MeCP2 expression are increased in excitatory synapses in the dLGN (Yagasaki et al., 2018). Dark rearing during this period results in the downregulation of MeCP2 protein. A study further shows that synaptic development is initially normal between *Mecp2* KO mice (Bird line) and their controls, but AMPAR-mediated synaptic transmission becomes weaker during later development (Noutel et al., 2011). MeCP2 levels in GABAergic interneurons are stable during development (Yagasaki et al., 2018), but its exact contribution to E/I balance remains undefined.

## Critical Elements That Contribute to Excitation/Inhibition Imbalance in Rett Syndrome

The loss of E/I balance can be caused by a direct impact of MeCP2 deficiency on the glutamatergic and GABAergic pathways. Many important elements in the brain have also been found to play profound secondary effects by influencing these excitatory and inhibitory activities. Among these elements, brain-derived neurotrophic factor (BDNF) is the most important growth factor that affects neurotransmission and neuronal intrinsic property. The role of BDNF has been received significant attention. I refer the reader to several past reviews (Autry and Monteggia, 2012; Katz, 2014; Li and Pozzo-Miller, 2014; Miranda-Lourenço et al., 2020). Furthermore, an inward rectifying K^+^ channel Kir4.1 that functions to maintain normal extracellular K^+^ levels is important for neuronal excitability (Della Vecchia et al., 2021). Expression of this channel has been found to be downregulated in *Mecp2* KO mice (Jaenisch line) (Kahanovitch et al., 2018); its role has been reviewed previously (Kahanovitch et al., 2019). Here, I will highlight several other important elements that indirectly contribute to E/I imbalance in RTT.

### KCC_2_/NKCC_1_ Ratio

The K-Cl cotransporter isoform 2 (KCC_2_) and Na-K-_2_Cl cotransporter isoform 1 (NKCC1) are two major cation-chloride cotransporters (CCCs) in the brain, which are responsible for extruding and accumulating Cl^–^, respectively (Watanabe and Fukuda, 2015). Maintaining Cl^–^ homeostasis is critical for setting the polarity and driving force of GABA_*A*_R-mediated currents. The KCC2/NKCC1 ratio modification during brain development is important to switch GABAergic neurotransmission from an excitatory to an inhibitory state (Maffei et al., 2017). Abnormal alteration of the KCC2/NKCC1 ratio would alter E/I balance and contribute to RTT neuropathology (Ip et al., 2018). RTT patients have reduced KCC2/NKCC1 ratio in the cerebrospinal fluid due to lower levels of KCC2 expression (Duarte et al., 2013). Consistently, the expression of KCC2 but not NKCC1 is reduced in postmortem tissues of the RTT brain, including the cerebral cortex and hippocampus (Hinz et al., 2019). Induced pluripotent stem cells (iPSCs) from RTT patients also show a reduction in KCC2 expression and thus a delayed polarity switch (Tang et al., 2016, 2019). These studies further demonstrate that MeCP2 directly regulates KCC2 transcription and that overexpression of KCC2 restores GABAergic activity. In *Mecp2* KO mouse models (Bird line), a consistent decrease in the KCC2/NKCC1 ratio was seen at different ages, which results in a positive shift of reversal potentials in pyramidal neurons (Banerjee et al., 2016; Lozovaya et al., 2019). The reduction appears to mainly occur in the frontal brain areas (Gigliucci et al., 2021). In contrast to these reports, a study in the cerebral cortex shows that KCC2 expression in adult *Mecp2* Het mice (Bird line) is generally normal with high variability although it is consistently low in presymptomatic mice (Oyarzabal et al., 2020). In a small sample of RTT patients, it is even higher in brain tissues. It was suggested that KCC2 may not be directly targeted by MeCP2 but rather a secondary effect of neuronal activity. A more recent report also demonstrates no change in total levels of KCC2 and NKCC1 in the hippocampus but shows altered phosphorylation levels of these two channels (Belaïdouni et al., 2021). It remains to be studied if the alteration of the KCC2/NKCC1 ratio in *Mecp2* KO mice is brain region-specific and the consequence of altered phosphorylated status on inhibitory neuronal activity.

### Cholinergic Activity

The cholinergic system has a crucial role in cognitive function and is affected in RTT individuals (Berger-Sweeney, 2003). Postmortem RTT brain tissues show fewer cholinergic cells, reduced choline acetyltransferase (ChAT) activity, and decreased cholinergic receptor expression (Wenk and Mobley, 1996; Wenk, 1997; Wenk and Hauss-Wegrzyniak, 1999; Brašić et al., 2012). *Mecp2* mutant animals also show reduced acetylcholine (ACh) and ChAT activity in the hippocampus, amygdala, striatum, thalamus, basal forebrain, and LC (Ricceri et al., 2011; Oginsky et al., 2014; Leung et al., 2017; Zhou et al., 2017; Murasawa et al., 2021). In *Mecp2* KO mice (Bird line), currents mediated by nicotinic ACh receptors (nAChRs) are smaller in LC neurons (Oginsky et al., 2014). However, the degree of the frequency change in sIPSPs is larger in *Mecp2* KO neurons during activation or inhibition of nAChRs, suggesting that nAChR modulation of GABAergic input is increased in *Mecp2* KO mice. This enhanced modulation by nAChRs does not occur in glutamatergic innervations on LC neurons. It was hypothesized that the machinery of GABA release is more sensitive due to possible changes of nAChR subunit composition in *Mecp2* KO interneurons. The nAChR subunits α7 and α2 have been shown to be influenced by MeCP_2_ deficiency (Zhang et al., 2016). Conditional deletion of *Mecp2* in basal forebrain cholinergic neurons results in a decreased expression of α7-containing nAChRs and reduced neuronal activity in the hippocampus. This nAChR subunit is mainly expressed in PV+ interneurons. Thus, decreased nAChR activity in interneurons causes a low GABAergic inhibition and a high glutamatergic excitation due to the disinhibition. Using the same strategy to delete *Mecp2* from cholinergic neurons in layers 5/6 of the perirhinal cortex leads to the loss of the highly variable firing pattern characteristic of WT neurons (Ballinger et al., 2019). It is unknown how excitatory and inhibitory activities are involved in this alteration. In the cholinergic interneurons of the nucleus accumbens (NAc), deletion of *Mecp2* results in reduced neuronal activity, which is mediated by the enhanced activity of α2-containing GABA_*A*_Rs (Zhang et al., 2020). Cholinergic modulation of glutamatergic and GABAergic neurons is clear, but its precise role in regulating E/I balance awaits additional work.

### Perineuronal Nets

Perineuronal nets (PNNs) are specialized extracellular matrix responsible for the closure of the critical period for developmental plasticity (Reichelt et al., 2019). An early study indicates overexpression of PNNs in the motor cortex of RTT girls (Belichenko et al., 1997). Consistent with the precocious maturation of PV+ interneurons, PNNs are consistently higher in *Mecp2* KO mice (Bird line) during the critical period (Krishnan et al., 2015; Krishnan et al., 2017; Patrizi et al., 2020). Using super-resolution imaging to better characterize PNNs, a group finds that PV+ interneurons in the visual cortex are surrounded by more premature and higher density of PNNs in *Mecp2* KO mice (Bird line) (Sigal et al., 2019). On the contrary, another study does not find increased inhibitory activity in the visual cortex and PNNs are also indistinguishable between WT and *Mecp2* KO mice (Bird line) (Banerjee et al., 2016). In the CDKL5 model of an RTT variant, the density but not the percentage of PV+ interneurons surrounded by PNNs is enhanced in the visual cortex of *CDKL5* KO mice, as compared with WT controls (Pizzo et al., 2016). Like the visual cortex, the density of PNNs in the somatosensory cortex is higher in *Mecp2* HET mice (Bird line) (Lau et al., 2020b). Maternal experience induces region-specific alteration of PNNs in WT but fails to do so in *Mecp2* HET mice. Elevated levels of PNNs are also seen in hippocampal CA2 of *Mecp2* KO mice (Bird line), which can be downregulated via chemogenetic reduction of neuronal activity (Carstens et al., 2021). This study further demonstrates that an extracellular matrix degrading enzyme matrix metalloproteinase (MMP) is reduced in *Mecp2* KO mice, which may explain the increased expression of PNNs.

## Cellular Consequences of Excitation/Inhibition Balance

Synaptic plasticity refers to the capacity of activity-dependent modification of synaptic strength (Citri and Malenka, 2008). To continually maintain such function, the brain is equipped with a negative feedback system for homeostatic regulation of network-wide neuronal activity (Turrigiano and Nelson, 2004). Proper glutamatergic and GABAergic synaptic transmission is foundational to establishing these two critical cellular events. I will discuss how E/I balance potentially contributes to the dysfunction of these cellular mechanisms in RTT patients and animal models.

### Short- and Long-Term Synaptic Plasticity

Short-term synaptic plasticity resulting from repetitive neuronal activity acts on a timescale of tens of milliseconds to a few minutes and normally involves presynaptic mechanisms (Zucker and Regehr, 2002). Considerable efforts have been made to evaluate several classical forms of short-term plasticity in *Mecp2* mutants, including paired pulse facilitation/depression (PPF/PPD), short-term depression (STD), and post-tetanic potentiation (PTP). An increase in the EPSP/EPSC magnitude (referred to as PPF) is mainly caused by residual Ca^2+^ that promotes the second response, whereas the decreased response (PPD) is generally due to inactivation of voltage-gate Ca^2+^ channels that inhibit transmitter release. In dissociated hippocampal neurons and acute slices, where the size of eEPSCs or field EPSPs is normally increased, PPF is decreased in MeCP2-deficient groups (Moretti et al., 2006; Nelson et al., 2006; Calfa et al., 2011; Nelson et al., 2011; Li et al., 2016). However, the decrease is also seen in synapses, where basal neurotransmission is normal (Asaka et al., 2006; Weng et al., 2011) or decreased (Gambino et al., 2010). Similarly, STD that typically reflects the depletion rate of readily releasable vesicles is also decreased during 10-Hz stimulation in *Mecp2* mutants (Nelson et al., 2006). In contrast to alterations of the fast action of presynaptic function, the relatively slow presynaptic activity that involves mobilization of the reserve pool of synaptic vesicles appears unaffected, as evidenced in unchanged PTP induced by high frequency stimuli (Asaka et al., 2006; Guy et al., 2007). These presynaptic measures appear to be normal in some models. For instance, intracellular whole-cell recordings revealed that PPF and/or STD are not affected by *Mecp2* deletion in autaptic hippocampal neurons and neocortical layer 5 pyramidal neurons (Chao et al., 2007; Dani and Nelson, 2009).

Long-term synaptic plasticity including long-term potentiation (LTP) and depression (LTD) has become a powerful parameter for understanding the neurobiological basis of RTT. MeCP2 deficiency in symptomatic mice results in impairments in LTP and LTD in hippocampal CA3–CA1 synapses (Jaenisch line) (Asaka et al., 2006). The impaired LTP and neurological abnormalities in *Mecp2* mutant mice can be reversed following *Mecp2* reactivation by genetic manipulation (Guy et al., 2007). Mechanistically, LTP defect has been associated with abnormally enhanced AMPAR-mediated synaptic transmission (Moretti et al., 2006). LTP defect is also shown in synapses with increased NMDAR activity; partial blockade by memantine restores the early phase of LTP (Weng et al., 2011). LTP in hippocampal CA1 generally requires AMPAR trafficking into the postsynaptic site (Diering and Huganir, 2018). In hippocampal CA1 of *Mecp2* KO mice (Jaenisch line) with higher synaptic transmission, LTP is occluded in these potentiated synapses because they fail to insert AMPARs into the membrane (Li et al., 2016). It should be noted that LTP defect may also be attributable to the reduced number of synapses. Paired recordings in cortical layer 5 pyramidal neuron of *Mecp2* KO mice (Jaenisch line) demonstrate LTP deficit, which can be restored when postsynaptic neurons are given sufficient depolarization to overcome weak connection (Dani and Nelson, 2009). It is also the case for cortico-lateral amygdala synapses where LTP is intact after paring pre- with postsynaptic activities (Gambino et al., 2010). Apart from glutamatergic activity, the role of proper GABAergic function is also important for synaptic plasticity. A report shows that LTP is impaired in a mouse model deficient of MeCP2 specifically in GABAergic interneurons (Chao et al., 2010). The impairment of LTP-like plasticity is also shown in the motor cortex of RTT patients, which favors cortical hyperexcitation as a result of GABAergic dysfunction (Bernardo et al., 2020). LTP in hippocampal CA2 of *Mecp2* KO mice (Bird line) is disrupted due to enhanced expression of PNNs and can be rescued by degrading PNNs (Carstens et al., 2021). LTD is also affected in CA3 pyramidal neurons of *Mecp2* KO mice (Bird line) because of the loss of developmental shift of GABAergic activity (Lozovaya et al., 2019). These findings indicate that maintaining E/I balance is critical for the induction of synaptic plasticity.

### Homeostatic Synaptic Plasticity

Homeostatic synaptic plasticity is accomplished by adjusting neuronal intrinsic or synaptic properties. Synaptic scaling up and down, two of the best-characterized forms of homeostatic plasticity, have been recently used in dissociated cultures or slices to determine the role of MeCP2. Synaptic scaling down during the bicuculline treatment is impaired in *Mecp2*-lacking hippocampal neurons (Qiu et al., 2012). In wildtype neurons, scaling down is mechanistically associated with MeCP2 upregulation and GluA2 receptor downregulation. Using a mouse model with *Mecp2* phosphate mutation at S421 and S424, a study further indicates that phosphorylation at these two loci is necessary for synaptic scaling down but not up (Zhong et al., 2012). This modification is mediated by metabotropic glutamate receptor 5 (mGluR5). Research on CNQX-eliciting scaling up demonstrates a loss of GluA1-mediated enhancement of spontaneous synaptic transmission in pyramidal neurons deficient in MeCP2 (Blackman et al., 2012). Furthermore, increased synaptic activity induced by visual deprivation is disrupted in *Mecp2* KO mice, which provides *in vivo* evidence for the role of MeCP2 in synaptic scaling up.

In a more comprehensive study, by monitoring GluA1 trafficking in and out of membrane, both scaling up and down were found to be impaired in *Mecp2* KO neurons treated with TTX and bicuculine, respectively. Early endosome antigen 1 (EEA1), a protein involved in GluA1 endocytosis, is lower in *Mecp2* KO mice (Jaenisch line) (Xu and Pozzo-Miller, 2017). Expression of EEA1 in *Mecp2* KO mice reduces the amplitude of mEPSCs and at the same time restores synaptic scaling down. These findings suggest that *Mecp2* has a role in maintaining both normal synaptic transmission and synaptic scaling, but future investigation is needed to explore shared mechanisms and the interplay between them.

### Pharmacological Treatments That Improve Excitation/Inhibition Balance

As E/I balance is of paramount importance in many brain events, restoration of E/I balance should be the aim for designing any treatments. In the last several decades, many genetic, pharmacological, and physiological treatments have been tested in individuals with RTT or mouse models, but few were assessed in the context of E/I balance. Here, I will discuss some pharmacological interventions for RTT and their potential impact on excitatory and/or inhibitory activities.

### Glutamatergic Modulators

In those brain regions where excitatory synaptic transmission is decreased, boosting AMPAR-mediated activity is likely to be beneficial. AMPAkines, positive allosteric AMPAR modulators, are known to increase the size of AMPAR-mediated excitatory synaptic responses and enhance BDNF expression (Lynch and Gall, 2006). Chronic treatment with the AMPAkine CX546 restores normal breathing patterns in *Mecp2* KO mice (Jaenisch line) (Ogier et al., 2007).

The role of NMDAR dysfunction in RTT has been evident in multiple brain regions (Blue et al., 1999, 2011; Durand et al., 2012; Nguyen et al., 2012; Mierau et al., 2016). Ketamine, an NMDAR antagonist, is known to enhance neuronal activity in the forebrain by disinhibiting cortical pyramidal neurons (Jackson et al., 2004). In the hypoactive forebrain of *Mecp2* KO mice (Jaenisch line), a sub-psychotomimetic dose of ketamine is able to restore neuronal activity and sensorimotor gating activity (Kron et al., 2012). In the visual cortex of *Mecp2* KO mice (Bird line), premature enhancement of GluN2A/GluN2B can also be inhibited by ketamine, leading to a delay of visual regression (Patrizi et al., 2016). However, since RTT brains demonstrate a mixture of hypo- and hyperactivity, the effect of ketamine on those neural circuits with higher activity is unknown. Furthermore, in the *CDKL5* KO mouse model of atypic RTT, the enhanced expression of GluN2B in the hippocampus accounts for seizures (Okuda et al., 2017). Treatment with the GluN2B-selective antagonist ifenprodil is capable of inhibiting epileptic activity.

In addition to these ionotropic glutamate receptors, metabotropic glutamate receptors (mGlu) are also affected in RTT. Postsynaptic mGlu5 is reduced in RTT patients and MeCP2-deficient mice (Bird line) (Gogliotti et al., 2016). Treatment with a mGlu5 modulator VU0462807 restores normal synaptic plasticity, motor behaviors, and other general phenotypes. The activation of presynaptic mGlu7 on interneurons is required for LTP in the hippocampal CA3–CA1 synapses (Klar et al., 2015). Its expression level is also low in RTT patients and mice (Gogliotti et al., 2017). Modulation of mGlu7 by VU0422288 leads to the restoration of respiratory, learning, and social deficits.

### GABAergic Modulators

Pharmacological modulation of GABAergic activity has shown to be effective in recovering cellular, network, behavioral dysfunction in RTT patients and mice (Braat and Kooy, 2015). In primary hippocampal neurons, *Mecp2* KD results in a decrease in the frequency of mEPSCs and an impairment in network activity, both of which can be reversed by a GABA_*A*_R modulator pentobarbital (Ma et al., 2015). In the respiratory center of the brainstem, the E/I ratio is generally increased. Enhancing GABAergic activity by the GABA reuptake blocker NO-711 or the GABA_*A*_R modulators benzodiazepines, produces a reduction of the number of apneas and an improvement in breathing rhythm in *Mecp2* KO mice (Bird line) (Abdala et al., 2010; Voituron and Hilaire, 2011). Administration of the GABAergic reuptake inhibitor tiagabine to *Mecp2* KO mice (Bird line) extends the life span (El-Khoury et al., 2014). In addition to synaptic GABA_*A*_Rs, activation of extrasynaptic GABA_*A*_Rs by THIP also rescues these abnormalities (Zhong et al., 2016). To avoid an excessive inhibition of the hypoactive forebrain, cloperastine that preferentially targets the brainstem was used to treat *Mecp2* KO mice (R168X line) (Johnson et al., 2020). It markedly decreases the occurrence of breathing irregulates. Mechanistically, it mainly excites GABAergic interneurons via blockade of Kir channel activity.

### Brain-Derived Neurotrophic Factor Mimetics

Brain-derived neurotrophic factor is a neurotrophic factor and has a profound role in modulating neurotransmission and plasticity of excitatory and inhibitory synapses (Schuman, 1999). BDNF levels are reduced in the entire brain of *Mecp2* KO mice (Jaenisch line) (Chang et al., 2006; Li et al., 2012); augmenting the BDNF signaling is a promising avenue to treat RTT (Li and Pozzo-Miller, 2014). Acute BDNF treatment reverses neuronal hyperexcitability in the brainstem nTS (Kline et al., 2010). To overcome its limited ability in crossing the blood–brain barrier, two BDNF mimetics LM22A-A and PTX-BD4-3 have been developed, which have a high penetration rate and selectively activate BDNF downstream signaling. Administration of LM22A-4 or PTX-BD4-3 to *Mecp2* HET mice (Jaenisch line) restores normal respiratory frequency, motor activity, and object location memory (Schmid et al., 2012; Li et al., 2017; Adams et al., 2020). Behavioral recovery is associated with the decrease of the hyperactive network and the reduction of the oversaturated excitatory synaptic transmission. Fingolimod, another BDNF mimetic that can stimulate BDNF expression, ameliorates symptoms in *Mecp2* KO mice (Bird line) (Deogracias et al., 2012), although its effectiveness in treating RTT patients was recently shown to be limited (Naegelin et al., 2021). A BDNF receptor activator 7,8-dihydroxyflavone was also reported to extend the life span and improve locomotor activity and breathing issues in *Mecp2* KO mice (Jaenisch line) (Johnson et al., 2012).

### Antidepressants

Many antidepressants have been used to treat RTT (Persico et al., 2019). Desipramine that functions to inhibit norepinephrine reuptake improves breathing rhythm and prolongs life span in *Mecp2* KO mice (Roux et al., 2007; Zanella et al., 2008). Mirtazapine, a more tolerable noradrenergic antidepressant also ameliorates motor and social behaviors in RTT patients and *Mecp2* KO mice (Bird line) (Bittolo et al., 2016; Flores Gutiérrez et al., 2020). Examination of pyramidal neurons in the somatosensory cortex reveals that mirtazapine restores neuronal dendritic arborization and spine density and improves glutamatergic and GABAergic activity in *Mecp2* KO mice. Fluoxetine, a serotonin reuptake inhibitor, can rescue motor abnormality by increasing the number of MeCP2+ cells in *Mecp2* HET mice (Bird line) (Villani et al., 2020, 2021). The molecular mechanisms underlying antidepressant treatments have been related to BDNF expression and phosphorylation of MeCP2. Chronic treatment with several major classes of antidepressants increases BDNF levels (Nibuya et al., 1995; Altar et al., 2003). Imipramine, an inhibitor of the serotonin and norepinephrine transporters, induces phosphorylation of MeCP2 at Ser421 (Hutchinson et al., 2012). The effect of antidepressant fluoxetine involves releasing MeCP2 from binding to *bdnf* promoter and enhancing BDNF expression (Jin et al., 2017). A recent study also shows an inverse relationship in which the effect of antidepressants is associated with phosphorylation of MeCP2 by released BDNF (Kim et al., 2021). Clearly, these antidepressants affect both excitatory and inhibitory neurotransmission, but their detailed role in modifying E/I balance remains to be discovered.

### Insulin-Like Growth Factor-1

Insulin-like growth factor-1, a neurotrophic factor, is critical for neuronal maturation and survival by activating PI3K-AKT-mTOR and MAPK-ERK pathways (Riikonen, 2016). Expression of IGF-1 and its downstream signals is downregulated in *Mecp2* KO and HET models (Jaenisch and Bird lines) (Ricciardi et al., 2011; Castro et al., 2014). Administration of IGF-1 peptide or recombinant full-length human IGF-1 (rhIGF-1) to RTT patients and mouse models has been shown to improve some symptoms and prolong life span, although its effectiveness is variable (Tropea et al., 2009; Castro et al., 2014; Khwaja et al., 2014; Pini et al., 2016; O’Leary et al., 2018; Yuan et al., 2020). Treatment with IGF-1 increases the amplitude of eEPSCs and the levels of PSD-95 in *Mecp2* KO mice (Bird line) (Tropea et al., 2009; Castro et al., 2014) or *CDKL5* KO mice (Della Sala et al., 2016). RTT patients’ iPSCs exhibit a low density of excitatory synapses and short neurite length, which can be normalized by IGF-1 treatment (Marchetto et al., 2010; de Souza et al., 2016). IGF-1 also improves network connectivity in MeCP2-deficient hippocampal neurons (Sun et al., 2018). Furthermore, IGF-1 or rhIGF-1 promotes the KCC2/NKCC1 ratio in the visual cortex (Banerjee et al., 2016; Gigliucci et al., 2021). These effects are beneficial to the improvement of the E/I balance.

### Histone Deacetylase 6 (HDAC6) Inhibitors

Histone deacetylase 6, a microtubule-associated enzyme, plays a negative role in cell migration and vesicle transport by regulating acetylation of α-tubulin (Hubbert et al., 2002). Increased levels of HDAC6 have been found in MeCP2-deficient cells, indicating the disturbance of microtubule dynamics (Delépine et al., 2015; Gold et al., 2015). An HDAC-6 inhibitor, tubastatin-A can correct microtubule defects and reverse impaired exploratory activity in *Mecp2* KO mice (308/Y line) (Lebrun et al., 2021). The treatment also enhances BDNF anterograde and retrograde transport and release (Xu and Pozzo-Miller, 2017), therefore allowing secreted BDNF to improve the E/I ratio. However, another study shows that inhibition of HDAC by a non-selective inhibitor, in turn, reduces the frequency of mEPSCs in control neurons to a level similar as seen in *Mecp2* KO neurons (Bird line) (Nelson et al., 2006). The exact mechanisms by which HDAC affects neuron activity need to be investigated.

### Mitochondria Targeting Drugs

Bacterial cytotoxic necrotizing factor 1 (CNF1) activates intracellular Rho-GTPases and protects mitochondria from the production of reactive oxygen species (ROS) (Travaglione et al., 2014; De Filippis et al., 2015b; Fabbri et al., 2018). Through these effects, CNF1 modulates actin cytoskeleton dynamics and enhances synaptic transmission and plasticity (Amir et al., 1999; Diana et al., 2007). *Mecp2* KO and HET mice treated with CNF1 show a significant improvement in cognition and motor function (308/Y and Bird line) (De Filippis et al., 2012; Urbinati et al., 2021). CNF1 treatment also prevents an enhancement of GluN2B-tyrosine phosphorylation and facilitates LTP induction in hippocampal CA1 of *Mecp2* HET mice. These findings imply that CNF-1 can promote E/I balance (De Filippis et al., 2015a).

Vitamin E is an important antioxidant that prevents oxidative damage by scavenging ROS (Lee and Han, 2018). Serum levels of vitamin E are lower in RTT patients (Formichi et al., 1998). Hippocampal slices treated with the vitamin E derivative, Trolox, show the dampening of hyperactive synaptic transmission and the improvement of short- and long-term synaptic plasticity (Janc and Müller, 2014). Systemic Trolox administration also ameliorates some synaptic and behavioral phenotypes in *Mecp2* KO mice (Bird line) (Janc et al., 2016). CoQ10 is a substance that also can attenuate ROS in RTT patients; however, its effect has not yet been determined (Di Pierro et al., 2020).

## Concluding Remarks

The E/I imbalance certainly is a hub of RTT pathogenesis, which manifests the outcome of genetic and molecular perturbations and underlies the progression trajectories of neurologic signs. Our understanding of E/I imbalance in RTT has made a great deal of progress. However, the evidence that we currently possess is mainly derived from assessments on either an excitatory or inhibitory side. In other words, we have not yet confidently explicated the role of E/I imbalance in an entire synaptic and circuit realm, nor fully elucidate the relevance of the loss of E/I stability in RTT symptoms and evaluate all potential therapies on the basis of the E/I outcome. With the increasing endeavors devoted to addressing the role of E/I imbalance in RTT, we hope that the scientific knowledge gleaned from these studies will be translated into effective therapies available for RTT and other neurological and psychiatric diseases that share similar neuropathological substrates.

## Author Contributions

WL wrote and edited the manuscript and approved the submitted version.

## Conflict of Interest

The author declares that the research was conducted in the absence of any commercial or financial relationships that could be construed as a potential conflict of interest.

## Publisher’s Note

All claims expressed in this article are solely those of the authors and do not necessarily represent those of their affiliated organizations, or those of the publisher, the editors and the reviewers. Any product that may be evaluated in this article, or claim that may be made by its manufacturer, is not guaranteed or endorsed by the publisher.
